# Growth Variation Among Thai Duckweed Species Under Axenic Conditions

**DOI:** 10.3390/biology15020159

**Published:** 2026-01-16

**Authors:** Siwaporn Jansantia, Yosapol Harnvanichvech, Athita Senayai, Nuttha Sanevas, Tokitaka Oyama, Ekaphan Kraichak

**Affiliations:** 1 Department of Botany, Faculty of Science, Kasetsart University, Bangkok 10900, Thailand; siwaporn.jans@ku.th (S.J.); yosapol.harn@ku.th (Y.H.); athita.s@ku.th (A.S.); nuttha.s@ku.th (N.S.); 2 Duckweed Holobiont Resource & Research Center (DHbRC), Kasetsart University, Bangkok 10900, Thailand; 3 Department of Botany, Graduate School of Science, Kyoto University, Kyoto 606-8502, Japan; oyama.tokitaka.8w@kyoto-u.ac.jp

**Keywords:** duckweed, relative growth rate, growth pattern

## Abstract

Duckweed has attracted considerable attention for its high protein content, rapid growth, and broad potential in biotechnological applications. Understanding key phenotypic traits is crucial for unlocking and maximizing this potential. While most studies on duckweed growth have been conducted under natural or non-sterile conditions, here we minimize environmental influences and focus on the genetic component of growth by assessing growth performance under axenic culture. In this study, we measured relative growth rate (RGR) in four duckweed species, *Landoltia punctata* (G. Mey.) Les & D. J. Crawford, *Lemna aequinoctialis* Welw., *Spirodela polyrhiza* (L.) Schleid., and *Wolffia globosa* (Roxb.) Hartog & Plas. collected from various natural locations across Thailand. A total of six to seven strains were tested for each species. The relative growth rates of studied species ranged from 0.012 day^−1^ in *S. polyrhiza* to 0.162 day^−1^ in *W. globosa*. Significant intraspecific variation was observed in *L. punctata*, *S. polyrhiza*, and *W. globosa*, with the coefficients of variation between 9.6 to 109.9 percent. Each strain showed distinct growth characteristics: Most displayed a steady growth pattern, whereas *W. globosa* showed exponential growth at Day 35 after the start of experiment. The results provide the first systematic comparisons of baseline growth rate data for duckweed species in Thailand. These findings advance the understanding of strain-specific growth traits in duckweed and establish a standardized protocol for evaluating growth traits under axenic conditions, providing a basis for future research and applications.

## Simple Summary

Duckweed is a small aquatic plant that can grow rapidly and produce high amounts of protein, making it applicable for various applications, including animal feed, wastewater treatment, and human food. To reach its full potential, we need to understand how different species of duckweed grow and whether their growth differs within the species. So far, studies have determined the growth in natural settings, making it difficult to separate the effects of the environment from genetics. In this study, we grew four duckweed species in the same sterile conditions. We obtained various strains from multiple locations in Thailand and measured how fast they grow. We found that strains within the same species grew at different rates, and different species had different growth patterns. The results provide the first systematic comparisons of baseline growth rate data for duckweed species in Thailand, as well as a standardized method for measuring growth under controlled conditions. This knowledge will allow researchers and industries to select the most productive strains for their respective applications.

## 1. Introduction

Duckweeds are free-floating aquatic angiosperms in the family Lemnaceae [1,2]. This family (Lemnaceae) consists of five genera, namely *Landoltia* Les & D. J. Crawford, *Lemna* L., *Spirodela* Schleid., *Wolffia* Horkel ex Schleid, and *Wolffiella* Heglm., comprising 35 recognized species and two interspecific hybrids worldwide [3,4]. Duckweeds are known for their rapid growth, simple structure, efficient nutrient uptake, and easy reproduction, making them valuable for animal feed [5,6], biofuel production [7,8], and sustainable food applications [9,10,11]. Recently, interest in duckweed has increased with the availability of complete genome sequences (e.g., *Spirodela polyrhiza*, *Lemna minor*, *Wolffia australiana*) [12,13,14,15] and the establishment of transformation protocols in species such as *Spirodela polyrhiza* [16], *Lemna aequinoctialis* [17], and *Lemna minor* [18], positioning duckweed as a promising platform for biotechnology. As both basic research and applied uses expand, a deeper understanding of duckweed diversity at the genetic and phenotypic levels will be essential to accelerate its effective utilization.

While many studies have explored intraspecific genetic diversity in duckweed species [19,20], intraspecific variation in phenotypes, such as growth rate, nutrient uptake, and stress responses, is less studied but is key to the effective application of duckweed species. For instance, Sree et al. [21] showed that strains of duckweed collected from different habitats varied significantly in salt tolerance, while Chen et al. [22] reported strain-specific differences in *Spirodela polyrhiza* under heavy metal (cadmium) exposure. These examples demonstrate that limited genetic divergence can nonetheless produce measurable physiological and ecological differences, highlighting the importance of intraspecific diversity for functional traits. However, the extent to which strain-specific genetic backgrounds explain growth variation, particularly under standardized axenic conditions where environmental influences are minimized, remains unclear.

Here, we establish a systematic approach to assess intraspecific phenotypic variation in growth performance among Thai duckweed species under axenic conditions, using colony proliferation and frond expansion as key parameters. Four species were examined, with each represented by six to seven strains: *Landoltia punctata* (G. Mey.) Les & D. J. Crawford, *Lemna aequinoctialis* Welw., *Spirodela polyrhiza* (L.) Schleid., and *Wolffia globosa* (Roxb.) Hartog & Plas. All strains were collected from diverse locations across Thailand and propagated from a single frond under axenic conditions in identical media to minimize environmental influences. Relative growth rates (RGR) were calculated from both the number of colonies and frond size expansion for each strain. We used analysis of covariance (ANCOVA) to test whether the relationship between colony number and frond size differed significantly among strains. In addition, an exponential fitting model was used to estimate divergence timing, providing a baseline for strain selection. Collectively, our findings provide a reference for ecotype strains in Thailand for future research and practical application, as well as a regional benchmark for evaluating growth traits under axenic conditions and support targeted uses of duckweed species.

## 2. Materials and Methods

### 2.1. Plant Collection and Preparation

Duckweed samples were collected from various locations throughout Thailand. The 27 samples from four species included six strains of *Landoltia punctata*, seven of *Lemna aequinoctialis*, seven of *Spirodela polyrhiza*, and seven of *Wolffia globosa* (Table S5). The specimens were identified to the species level, using the methods outlined in a previous study on Thai duckweed species [20]. All strains were surface sterilized under aseptic conditions to remove surface microorganisms. Fronds were immersed in 3% (*w*/*w*) sodium hypochlorite solution (available chlorine 6% *w*/*w*) for one minute, then rinsed twice with sterile distilled water for 30 s each. Sterilized fronds were subsequently cultured in NF medium [23]. Each sterilized sample was clonally propagated from a single frond to ensure genetic uniformity and maintained in a growth chamber under controlled conditions at 100 ± 10 μmol m^−2^ s^−1^ with an 8 h light/16 h dark photoperiod.

### 2.2. Growth Experiment

A single frond from each strain of *Landoltia punctata*, *Spirodela polyrhiza*, and *Lemna aequinoctialis* was cultured in Petri dishes (40 mm diameter × 12 mm height) containing 4 mL of NF culture medium. *Wolffia globosa* was cultured in 12-well plates, with each well (21.9 mm diameter × 17.5 mm height) containing 4 mL of NF medium. All cultures were maintained under the controlled conditions described above, and plates were periodically randomized on the growth shelves to minimize positional bias. In this study, we used the term “colony” to represent a set of connected fronds. An individual daughter colony was recognized when the daughter frond was completely detached from the mother frond, as observed from the photographs taken under a stereomicroscope, to avoid disturbance to the plant population. The size and number of colonies were recorded on days 0, 3, 7, 10, 14, 17, 21, 24, 28, 31, and 35. The relative growth rate (RGR) was calculated for the period from day 0 to day 21, as some cultures had fully expanded to occupy the available surface area by this time point. RGR was determined using the formula:

(1) $$RGR=\frac{\ln\left(N_{21}\right)-\ln\left(N_{1}\right)}{21}$$

where N_21_ is the number of colonies at Day 21, and N_1_ is the number of colonies at Day 1, regardless of whether the population had reached the stationary phase.

In addition, the doubling time (T_d_) was calculated over 35 days of the cultivation period, using the formula:

(2) $$T_{d}=\frac{\ln(2)}{\mathrm{RGR}}$$

### 2.3. Intraspecific Variation in Growth Rates

All descriptive statistics and statistical analyses were performed in R version 4.3.3 [24] with statistical significance set at *p* ≤ 0.05. Intraspecific variation was analyzed using Analysis of Variance (ANOVA). Prior to parametric testing, the assumptions of normality and homogeneity of variances were assessed using the Shapiro–Wilk test and Bartlett test, respectively. When parametric assumptions were not met, group comparisons were conducted using the non-parametric Kruskal–Wallis test. An Analysis of Covariance (ANCOVA) was also performed between colony size and strains, using the number of colonies as a covariate to determine different growth allocation strategies among the observed strains.

### 2.4. Quantification of Growth Divergence Timing

Growth divergence was calculated as the daily difference between the maximum and minimum mean colony numbers. To allow cross-species comparisons, each value was normalized to the maximum observed divergence, yielding the proportion of maximum divergence. An exponential model was fitted to the relationship between the proportion of maximum divergence and time using the “nls” function [24]. The best-fit model was evaluated through an iterative optimization process that minimizes the Residual Sum of Squares (RSS). Time points corresponding to defined proportions of divergence were then estimated by numerical root-finding with the “uniroot” function [24].

## 3. Results

### 3.1. Species Variations in Frond Size and Number of Colonies

To evaluate interspecific variation in Thai duckweed, we estimated mean relative growth rates (RGR) and doubling times from frond size expansion and number of colonies across four species (Tables S1 and S2). The RGRs based on colony size ranged from 0.028 to 0.151 day^−1^, while those based on the number of colonies ranged from 0.033–0.165 day^−1^. Corresponding doubling times varied between 4.63–28.42 days based on frond size and 4.243 to 14.428 days based on the number of colonies. Clear differences were observed among species, and both metrics revealed consistent interspecific variation (ANOVA, *p* < 0.05). The fastest-growing species was *Wolffia globosa*, with RGRs of 0.151 day^−1^ based on frond size and 0.165 day^−1^ based on number of colonies. In contrast, *Spirodela polyrhiza* exhibited the slowest growth, with rates of 0.028 day^−1^ based on frond size and 0.033 day^−1^ based on the number of colonies.

### 3.2. Strain Variation in Frond Size and Number of Colonies

**Figure 1:**
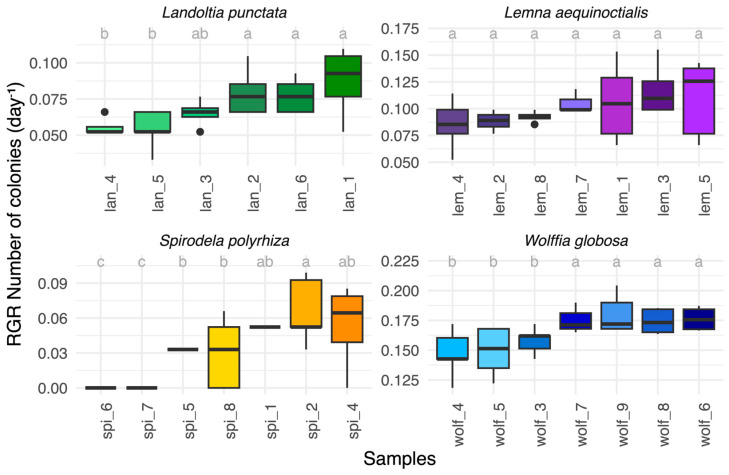
Strain-level variation in relative growth rate (RGR) based on the number of colonies in four duckweed species. Boxplots show RGR for individual strains; letters indicate significant differences (Tukey’s HSD, *p* < 0.05). Consistent with RGR based on frond size (Figure S1), *Spirodela polyrhiza* showed the lowest and most variable growth, *Wolffia globosa* the highest and most uniform, while *Landoltia punctata* and *Lemna aequinoctialis* were intermediate.

To assess strain-level variation, we examined relative growth rates (RGR) based on frond size expansion and colony number in 27 strains representing four duckweed species collected from diverse provinces in Thailand (Tables S1 and S2). All species exhibited different magnitudes of intra-specific variation in growth rates (Figure 1, Figures S1 and S2).

In *Landoltia punctata*, RGR based on frond size expansion ranged from 0.016 ± 0.002 day^−1^ in lan_4 from Pathum Thani to 0.079 ± 0.039 day^−1^ in lan_1 from Phayao, with the CV of 60% (Figure S1, Table S1). For the number of colonies, RGRs ranged from 0.053 ± 0.006 day^−1^ in lan_4 from Phitsanulok to 0.087 ± 0.023 day^−1^ in lan_1 from Phayao, with the CV of 26% (Figure 1, Table S2).

In *Lemna aequinoctialis,* RGR based on frond size expansion ranged from 0.059 ± 0.019 day^−1^ in lem_4 from Bangkok to 0.115 ± 0.010 day^−1^ in lem_3 from Pathum Thani, with the CV of 37% (Figure S1, Table S1). For the number of colonies, RGRs ranged from 0.085 ± 0.023 day^−1^ in lem_4 to 0.117 ± 0.023 day^−1^ in lem_3, with the CV of 25% (Figure 1, Table S2).

In *Spirodela polyrhiza*, RGRs were the lowest overall. RGR based on frond size expansion ranged from 0.012 day^−1^ in spi_5 from Nakhon Ratchasima and spi_7 from Bangkok to 0.067 ± 0.039 day^−1^ in spi_4 from Maha Sarakham, with a CV of 100% (Figure S1, Table S1). For number of colonies, RGRs ranged from 0.000 day^−1^ in spi_6 from Uthai Thani and spi_7 to 0.053 ± 0.038 day^−1^ in spi_4 with CV of 94%, indicating that spi_6 and spi_7 did not proliferate (Figure 1, Table S2)

In *Wolffia globosa*, RGRs were the highest and most uniform. RGR based on frond size expansion ranged from 0.142 ± 0.022 day^−1^ in wolf_4 from Bangkok and wolf_8 from Suphan Buri to 0.162 ± 0.005 day^−1^ in wolf_6 from Bangkok (Figure S1, Table S1), with a CV of 10%. For the number of colonies, RGRs ranged from 0.147 ± 0.020 day^−1^ in wolf_4 to 0.180 ± 0.016 day^−1^ in wolf_9 from Bangkok, with a CV of 11% (Figure 1, Table S2).

Consistent across all growth parameters, *S. polyrhiza* exhibited the greatest variation. *Landoltia punctata* and *Lemna aequinoctialis* showed intermediate variability, and *W. globosa* displayed the highest uniformity among strains (Figure 1, Figure S1). Moreover, CV values were consistently lower when calculated from the number of colonies than from frond size expansion, suggesting that colony number provides a more stable measure of intraspecific growth variation. Therefore, the subsequent report of the growth parameter focuses on the number of colonies as a primary parameter.

### 3.3. Strain Variation in Growth Allocation

**Figure 2:**
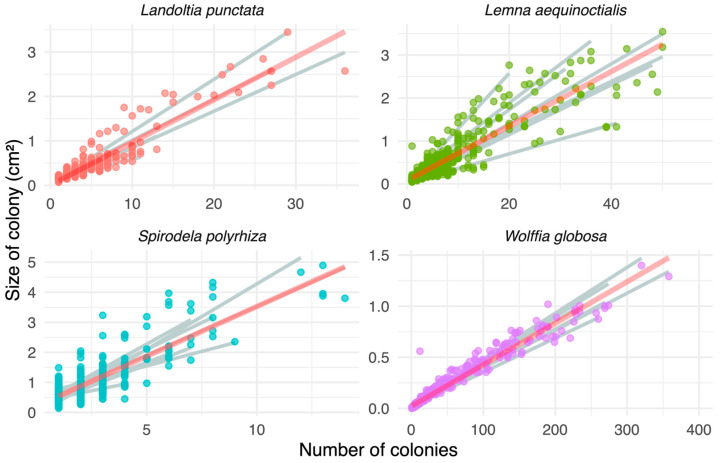
Regression between frond size expansion and the number of colonies across four duckweed species. The red line is the regression line for all data, while the grey lines represent individual regression lines for individual strains.

To understand how duckweed strains balance expansion (colony size) and proliferation (colony number), we analyzed the effects of colony number, strain identity, and their interaction on colony size using analysis of covariance (ANCOVA; Figure 2). The results revealed significant effects of colony number (*p* < 0.001), strain identity (*p* < 0.001), and their interaction (*p* < 0.001; Table S3) across all four duckweed species. Colony size increased significantly with colony number in all studied strains, indicating no tradeoff between size expansion and proliferation in duckweed. This positive relationship suggests that colony number serves as a reliable proxy for overall duckweed growth. However, significant strain effects and a strain-by-colony number interactions, reflected in differences in the slopes of the colony size-colony number relationships, indicate distinct growth strategies among strains within each species.

### 3.4. Growth Curve Pattern in Duckweed Strains

**Figure 3:**
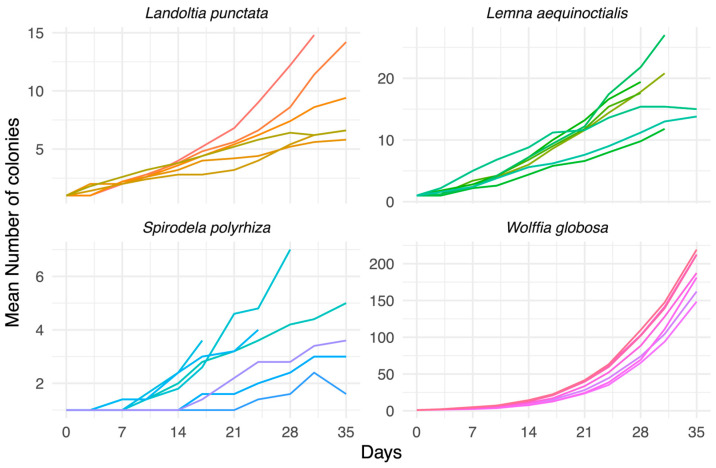
Mean number of colonies over time of different strains of four duckweed species. Each line represents the average values from multiple replicates for each strain over the period of experiment (Day 0 to Day 35).

To investigate how strain-level differences influence the dynamics of duckweed proliferation, we examined growth trajectories over a 35-day cultivation period. We found that the number of colonies increased in all species, but distinct trajectories were evident among genera and strains (Figure 3).

*Landoltia punctata* reached a maximum of approximately 15 colonies, with exponential growth typically initiated after day 7. Some strains plateaued early, whereas others continued increasing throughout the cultivation period.

*Lemna aequinoctialis* attained colony counts of up to about 25, with exponential growth evident between days 7 and 10. Strains differed in whether they entered the stationary phase or sustained growth until day 35.

*Spirodela polyrhiza* exhibited the slowest proliferation, with most strains producing fewer than six colonies by day 35. Exponential growth was delayed, generally beginning after day 14, and several strains plateaued before the end of the experiment. In contrast, *Wolffia globosa* demonstrated the most rapid proliferation, with exponential growth evident by day 10 and sharply accelerating thereafter.

All *Wolffia* strains exceeded nearly 200 colonies by day 35, with no indication of stationary-phase entry. Together, these trajectories demonstrate clear differences in growth dynamics, with *S. polyrhiza* proliferating slowest and *W. globosa* fastest among the four species.

### 3.5. Quantifying Divergence Timing Across Duckweed Species

**Figure 4:**
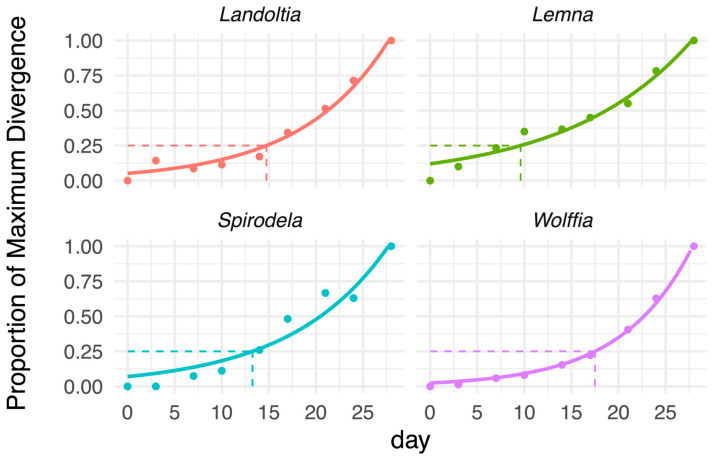
Divergence in colony number among Thai duckweed strains over time. Solid curves represent exponential model fits. Horizontal dashed lines mark 25% of maximum divergence, and vertical dashed lines indicate the estimated day when strains reached this threshold.

To identify when divergence among strains emerged, we estimated the time at which each species reached 25% of its maximum divergence using exponential model fitting (Figure 4; Table S4). Divergence became apparent earliest in Lemna, which reached 25% of its maximum divergence by day 10. *Spirodela* and *Landoltia* diverged later at days 14 and 15, respectively. *Wolffia* showed the slowest onset of divergence, with 25% reached only after day 18 (Figure 4). These results indicate that although early-phase growth was comparable across strains, the growth divergence became evident after approximately two weeks, with *Lemna* separating earliest and *Wolffia* latest.

## 4. Discussion

### 4.1. Growth Variability and Genetic Diversity of Duckweed

Our results revealed clear species-level differences in growth, with *Wolffia globosa* showing the fastest and most uniform proliferation, *Lemna aequinoctialis* and *Landoltia punctata* occupying intermediate positions, and *Spirodela polyrhiza* the slowest. At the strain level, variability in growth, as measured by the coefficient of variation (CV), was lowest in *W. globosa*, moderate in *Lemna aequinoctialis* and *Landoltia punctata*, and highest in *S. polyrhiza*. These phenotypic patterns align with genetic analyses of Thai duckweed populations based on three chloroplast markers (*rbcL*, *atpF–atpH*, *psbK–psbI*) and Tajima’s D values [20]. In Thailand, *Lemna aequinoctialis* and *S. polyrhiza* exhibited stronger genetic structuring, higher polymorphism, and significant negative Tajima’s D values, consistent with broader strain-level divergence in growth. By contrast, *W. globosa* and *L. punctata* showed more uniform growth, reflected in their relatively stable growth performance.

In contrast, analyses of Chinese populations revealed the opposite trend, with *W. globosa* and *Lemna aequinoctialis* exhibiting the highest genetic diversity, whereas *S. polyrhiza* and *Landoltia punctata* displayed very low levels of variation [25]. Collectively, we suggest that genetic variation in duckweed is closely linked to growth traits within Thai populations and potentially shaped by geography and demographic history across regions. Additional genetic markers, such as nuclear tubulin protein and ploidy level [19], could offer better resolution in detecting population structures that closely align with physiological differences.

### 4.2. Growth Variability and Growth Strategies of Duckweed

Relative growth rates (RGR) were estimated using two complementary parameters: number of colonies (Figure 1, Table S2) and frond expansion (Figure S1, Table S1). While both metrics captured inter- and intraspecific variation, their consistency differed. Coefficients of variation (CV) were generally lower for colony-based estimates than for frond-based measurements, with values of 26% versus 60% in *Landoltia punctata*, 25% versus 37% in *Lemna aequinoctialis*, and 94% versus 110% in *Spirodela polyrhiza*. In *Wolffia globosa*, both parameters showed similarly low variability (11% versus 9%) (Tables S1 and S2). These results indicate that colony-based RGR provides more consistent and reliable measures of duckweed growth studies.

Notably, *W. globosa* demonstrated the highest growth rate with the lowest variability, while *S. polyrhiza* showed the slowest proliferation and the greatest variation in growth rate. These differences in growth strategies among the studied species align with their known biology. Smaller species, such as *Wolffia* and *Lemna*, exhibited consistently rapid growth with low variability, suggesting limited plasticity in growth traits. This characteristic makes them well-suited for cultivation in stable, optimized environments [26]. Conversely, larger species, such as *Landoltia* and *Spirodela*, displayed greater variation in growth traits and generally slower growth rates. This pattern suggests high plasticity among genotypes, potentially enabling these species to tolerate a broader range of environmental conditions [27,28].

Several studies have examined the adaptive plasticity of individual duckweed species under metal stress [29,30], nutrient limitation [31], nitrogen limitation [32], and salt stress [33]. The available evidence nonetheless supports that growth variability and adaptive flexibility differ among species, which is consistent with the patterns observed in our study. For instance, Rzodkiewicz and Turcotte [34] showed that *L. minor* was more sensitive to ecotoxicants than *S. polyrhiza*, while Ruamsin et al. [30] reported that *Lemna aequinoctialis* was more susceptible to mercury toxicity than *S. polyrhiza*. These findings highlight that slower-growing taxa with high variability, such as *Spirodela*, may tolerate a broader range of environmental fluctuations, whereas fast-growing species, such as *Lemna,* exhibit more uniform responses under stable conditions. However, the link between genetic diversity and variation in growth strategies for duckweed remain unclear and require further investigation.

Together, these patterns point to a tradeoff between growth performance and plasticity. Species with high growth potential and low variability, such as *Wolffia* and *Lemna*, appear to represent more stable phenotypes optimized for rapid proliferation under favorable conditions. In contrast, slower-growing taxa like *Spirodela* exhibit greater variability, which may enable broader adaptive responses and confer resilience in fluctuating environments. We thus hypothesize that growth variability in duckweed plays an important role in shaping resilience and adaptive strategies.

### 4.3. Axenic Systems as Controlled Baselines for Genetic Variation

Previous studies have shown that duckweeds maintained in non-axenic cultures proliferate much faster than those under axenic conditions. Reported values suggest that most species typically double within 1–5 days (Table S6) [35], whereas in our axenic system, doubling times extended to 4–14 days (Tables S1 and S2). This contrast highlights the difference between axenic and non-axenic conditions. One explanation is the absence of microbial partners. Duckweed growth is strongly influenced by its microbiome. Ishizawa et al. [36] reported that co-cultivation of *Lemna* with environmental bacteria increased growth performance by about 14%. Another study in *Spirodela* showed even stronger effects with the increases of 226–317% when duckweed was associated with specific bacterial strains [37]. These bacteria enhance duckweed growth through functions such as nitrogen cycling, phosphorus solubilization, and auxin production. In their absence, growth is consistently reduced, underscoring the microbiome as a critical component of duckweed growth capacity.

In addition, the natural habitat of duckweeds is typically shallow, aerated ponds where continuous gas exchange, light [38], and nutrients [9] supports rapid proliferation. In contrast, our axenic cultures were maintained in closed systems, where gas exchange was restricted, particularly limiting the availability of CO_2_. Elevated CO_2_ has been shown to enhance growth and starch accumulation in *Lemna* [39,40], underscoring the importance of carbon supply for sustaining rapid proliferation. Thus, restricted aeration and limited CO_2_ availability in sealed systems are likely additional factors that constrain growth in our experiments. Future studies could address these constraints by incorporating controlled aeration, CO_2_ supplementation, or semi-open culture systems to better approximate natural conditions while maintaining sterility.

Although axenic conditions yield lower absolute growth rates, they maintain stable species rankings and enable reliable estimation of growth performance. By reducing variability and establishing consistent baselines, axenic systems facilitate more precise detection of genetic differences, microbial effects, and environmental influences. Therefore, axenic growth can serve as a distinct, measurable trait for duckweed growth study. Our experimental system thus represents a valuable platform for disentangling phenotypic variation from environmental effects and for establishing standardized conditions for strain selection. Our growth data also provide a standardized, axenic benchmark for a regional collection of strains, which enables precise future comparisons and strain selection for applied work.

## 5. Conclusions

We provided a comprehensive analysis of four duckweed species and 27 strains collected across Thailand under axenic conditions. Clear differences in growth rates and proliferation patterns were observed among species, with additional variability detected at the strain level. Overall, growth rates were several-fold lower than those typically reported under non-axenic conditions, underscoring the essential role of the microbiome in supporting duckweed performance. By applying standardized cultivation and evaluation methods, we demonstrated that strain-level differences can be reliably detected within two weeks. This framework establishes a practical benchmark for assessing duckweed growth, facilitating both ecological investigations and the strategic selection of species and strains for biotechnological applications. In addition, the concordance observed between growth variation and reported genetic diversity in Thai Duckweed suggests that quantitative traits in duckweed may be genetically tractable. Our standardized protocol can help establish the relationship between the diversity in growth strategies with genetic diversity in the future.

## Data Availability

Dataset available on request from the authors.

## Supplementary Materials

The following supporting information can be downloaded at: https://www.mdpi.com/article/10.3390/biology15020159/s1, Figure S1. Strain-level variation in relative growth rate (RGR, day^−1^) based on frond size expansion across four duckweed species. Boxplots show individual strains; letters denote significant differences (Tukey’s HSD, *p* < 0.05). *Spirodela polyrhiza* had the lowest and most variable growth, *Wolffia globosa* the highest and most uniform, while *Landoltia punctata* and *Lemna aequinoctialis* were intermediate; Figure S2. Photographs for duckweed species in the current study: A *Landoltia punctata* (G. Mey.) Les & D. J. Crawford, B *Lemna aequinoctialis* Welw., C *Spirodela polyrhiza* (L.) Schleid., and D *Wolffia globosa* (Roxb.) Hartog & Plas.; Table S1. Relative growth rate (RGR) and doubling time of Thai strains of four duckweed species by the frond size. Coefficient of variation (CV) and *p*-values from Kruskal-Wallis Test were calculated from the comparisons among the strain of the same species. Asterisks (*) indicate significant intraspecific variation; Table S2. Relative growth rate (RGR) and doubling time of Thai strains of four duckweed species by the number of colonies. Coefficient of variation (CV) and p-values from Kruskal-Wallis Test were calculated from the comparisons among the strain of the same species. Asterisks (*) indicate significant intraspecific variation; Table S3. ANCOVA results showing effects of colony number, strain identity, and their interaction on frond size expansion across duckweed species; Table S4. The number of days to reach 25% and 50% of maximum divergence in the number of colonies among the Thai strains of duckweed species; Table S5. Sampling information for duckweed strains used in this study. The table lists species, strain codes, collection sites, geographic locations (province, district), and GPS coordinates of sampling points. Table S6. Published data on duckweed growth traits. Relative growth rate (RGR, day^−1^) and doubling time (days) of selected strains of *Landoltia punctata*, *Lemna aequinoctialis*, *Spirodela polyrhiza*, and *Wolffia globosa* from different geographic origins, as reported by Ziegler et al. (2015) [35].
