# Supplementary material for: Growth Variation Among Thai Duckweed Species Under Axenic Conditions
Source: Biology (Basel). 2026 Jan 16;15(2):159. doi: 10.3390/biology15020159 (PMC12837684; doi:10.3390/biology15020159)
Supplement: Supplementary file 1 [file biology-15-00159-s001.zip › biology-4085062-supplementary.pdf]

Supplementary Materials

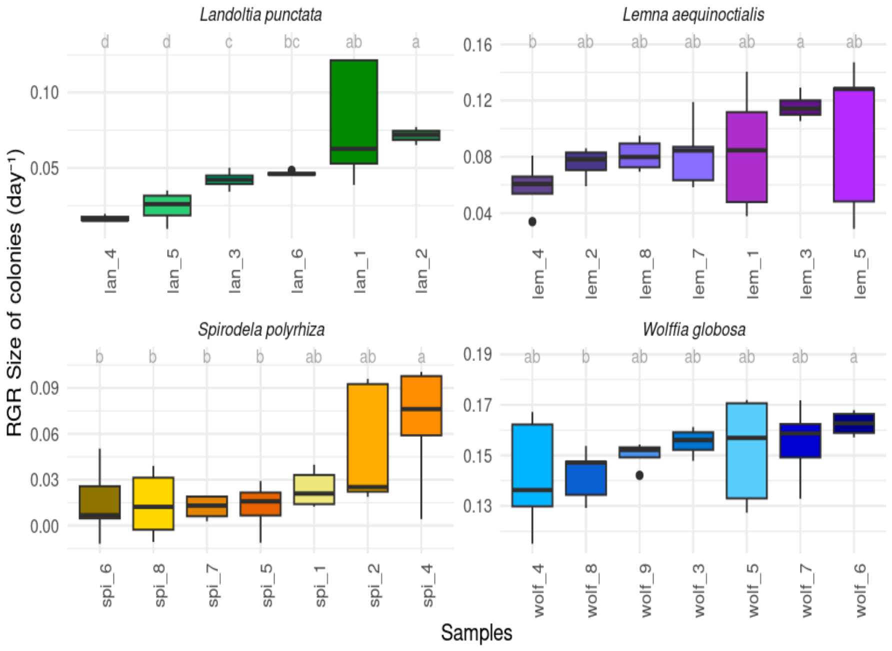

Figure S1. Strain-level variation in relative growth rate (RGR, day^{-1}) based on frond size expansion across four duckweed species. Boxplots show individual strains; letters denote significant differences (Tukey's HSD, $p < 0.05$). *Spirodela polyrhiza* had the lowest and most variable growth, *Wolffia globosa* the highest and most uniform, while *Landoltia punctata* and *Lemna aequinoctialis* were intermediate.

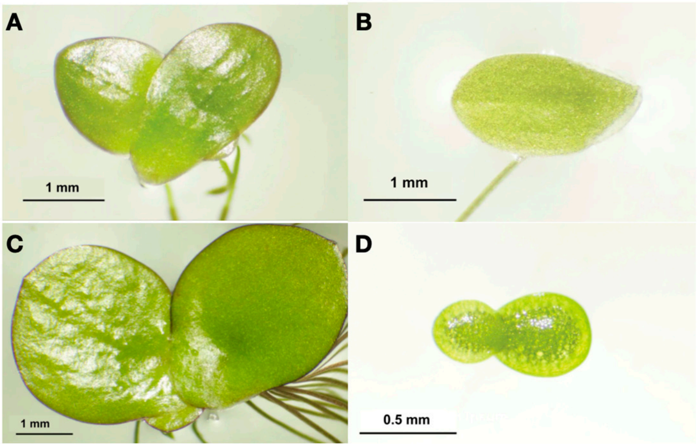

Figure S2. Photographs for duckweed species in the current study: **A** *Landoltia punctata* (G. Mey.) Les & D.J.Crawford, **B** *Lemna aequinoctialis* Welw., **C** *Spirodela polyrhiza* (L.) Schleid., and **D** *Wolffia globosa* (Roxb.) Hartog & Plas.

Table S1. Relative growth rate (RGR) and doubling time of Thai strains of four duckweed species by the frond size. Coefficient of variation (CV) and p-values from Kruskal-Wallis Test were calculated from the comparisons among the strain of the same species. Asterisks (*) indicate significant intraspecific variation.

Species	Number of strains	RGR (day^{-1})	Doubling Time (days)	CV (%)	P-value
Landoltia punctata	6	0.048 ± 0.029	20.98 ± 15.67	60.369	0.003*
lan_1		0.079 ± 0.039	10.69 ± 5.179		
lan_2		0.071 ± 0.006	9.74 ± 0.833		
lan_3		0.042 ± 0.006	16.74 ± 2.656		
lan_4		0.016 ± 0.002	41.96 ± 5.720		
lan_5		0.024 ± 0.011	36.77 ± 24.276		
lan_6		0.046 ± 0.001	14.99 ± 0.486		
Lemna aequinoctialis	7	0.085 ± 0.031	9.61 ± 4.56	37.229	0.275
lem_1		0.084 ± 0.043	10.43 ± 5.743		
lem_2		0.075 ± 0.011	9.384 ± 1.652		
lem_3		0.115 ± 0.010	6.019 ± 0.512		
lem_4		0.059 ± 0.019	12.955 ± 5.159		
lem_5		0.096 ± 0.053	10.780 ± 8.422		
lem_7		0.082 ± 0.023	8.958 ± 2.437		
lem_8		0.081 ± 0.010	8.649 ± 1.144		
Spirodela polyrhiza	7	0.028 ± 0.031	28.42 ±79.04	109.882	0.141
spi_1		0.024 ± 0.011	35.210 ± 16.831		
spi_2		0.050 ± 0.039	22.022 ± 13.786		
spi_4		0.067 ± 0.039	39.118 ± 68.035		
spi_5		0.012 ± 0.017	13.227 ± 51.796		
spi_6		0.015 ± 0.023	45.729 ± 79.383		
spi_7		0.012 ± 0.008	101.504 ± 95.128		
Wolffia globosa	7	0.151 ± 0.014	4.63 ± 0.48	9.658	0.294
wolf_3		0.155 ± 0.005	4.468 ± 0.171		
wolf_4		0.142 ± 0.022	4.973 ± 0.779		
wolf_5		0.151 ± 0.020	4.632 ± 0.656		
wolf_6		0.162 ± 0.005	4.265 ± 0.134		
wolf_7		0.155 ± 0.013	4.494 ± 0.421		
wolf_8		0.142 ± 0.010	4.887 ± 0.357		
wolf_9		0.150 ± 0.005	4.619 ± 0.174		

Table S2. Relative growth rate (RGR) and doubling time of Thai strains of four duckweed species by the number of colonies. Coefficient of variation (CV) and p-values from Kruskal-Wallis Test were calculated from the comparisons among the strain of the same species. Asterisks (*) indicate significant intraspecific variation.

Species	Number of strains	RGR (day^{-1})	Doubling Time (days)	CV (%)	P-value
Landoltia punctata	6	0.070 ± 0.018	10.542 ± 3.034	26.366	0.020*
lan_1		0.087 ± 0.023	8.544 ± 2.834		
lan_2		0.079 ± 0.016	8.958 ± 1.650		
lan_3		0.065 ± 0.009	10.823 ± 1.756		
lan_4		0.055 ± 0.006	12.562 ± 1.374		
lan_5		0.053 ± 0.013	13.699 ± 4.306		
lan_6		0.077 ± 0.011	9.129 ± 1.368		
Lemna aequinoctialis	7	0.101 ± 0.025	7.303 ± 1.938	25.199	0.346
lem_1		0.105 ± 0.036	7.213 ± 2.506		
lem_2		0.088 ± 0.009	7.912 ± 0.884		
lem_3		0.117 ± 0.023	6.060 ± 1.080		
lem_4		0.085 ± 0.023	8.697 ± 2.781		
lem_5		0.109 ± 0.035	6.990 ± 2.601		
lem_7		0.105 ± 0.011	6.619 ± 0.659		
lem_8		0.092 ± 0.005	7.521 ± 0.461		
Spirodela polyrhiza	7	0.033 ± 0.031	14.428±5.043	94.125	0.004*
spi_1		0.052 ± 0.000	13.249 ± 0.000		
spi_2		0.065 ± 0.028	12.395 ± 5.673		
spi_4		0.053 ± 0.038	10.139 ± 2.732		
spi_5		0.033 ± 0.000	21.000 ± 0.000		
spi_6		0.000 ± 0.000	N/A		
spi_7		0.000 ± 0.000	N/A		
spi_8		0.030 ± 0.030	14.916 ± 5.444		
Wolffia globosa	7	0.165 ± 0.018	4.243 ± 0.537	11.308	0.013*
wolf_3		0.157 ± 0.011	4.405 ± 0.319		
wolf_4		0.147 ± 0.020	4.785 ± 0.697		
wolf_5		0.148 ± 0.020	4.729 ± 0.672		
wolf_6		0.176 ± 0.010	3.943 ± 0.235		
wolf_7		0.174 ± 0.009	3.976 ± 0.219		
wolf_8		0.174 ± 0.010	3.988 ± 0.235		
wolf_9		0.180 ± 0.016	3.865 ± 0.329		

Table S3 ANCOVA results showing effects of colony number, strain identity, and their interaction on frond size expansion across duckweed species.

Species	Number of Colonies		Strain		Number of Colonies:Strain	
	F	P	F	P	F	P
Landoltia punctata	3173.337	$2.2x10^{-16}$	26.024	$2.2x10^{-16}$	23.284	$2.2x10^{-16}$
Lemna aequinoctialis	6046.418	$2.2x10^{-16}$	85.078	$2.2x10^{-16}$	65.683	$2.2x10^{-16}$
Spirodela polyrhiza	1247.590	$2.2x10^{-16}$	5.1669	$4.1x10^{-5}$	9.039	$3.2\ x10^{-9}$
Wolffia globosa	14256.618	$2.2x10^{-16}$	17.975	$2.2x10^{-16}$	22.314	$2.2x10^{-16}$

Table S4 The number of days to reach 25% and 50% of maximum divergence in the number of colonies among the Thai strains of duckweed species

Species	Day at 25% of Maximum Divergence	Day at 50% of Maximum Divergence
Landoltia punctata	14.74	21.23
Lemna aequinoctialis	9.61	18.70
Spirodela polyrhiza	13.24	20.48
Wolffia globosa	17.54	22.69

Table S5 Sampling information for duckweed strains used in this study. The table lists species, strain codes, collection sites, geographic locations (province, district), and GPS coordinates of sampling points.

Species	Samples	Province	Location (°N, °E)
Landoltia punctata	lan_1	Phayao	19.158611, 99.86056
	lan_2	Nakhon Pathom	14.011917, 99.97042
	lan_3	Suratthani	9.073778, 99.32767
	lan_4	Pathum Thani	14.032500, 100.72972
	lan_5	Phitsanulok	16.831819, 100.21202
	lan_6	Amnat Charoen	15.867103, 104.62151
Lemna aequinoctialis	lem_1	Bangkok	13.844444, 100.57083
	lem_2	Nakhon Ratchasima	14.723083, 102.02447
	lem_3	Pathum Thani	14.032500, 100.72972
	lem_4	Bangkok	13.851139, 100.73978
	lem_5	Samut Songkhram	13.470556, 99.97000
	lem_7	Lop Buri	14.924861, 100.90022
	lem_8	Ang Thong	14.602806, 100.42883
Spirodela polyrhiza	spi_1	Ang Thong	14.602806, 100.42883
	spi_2	Amnat Charoen	15.891297, 104.62262
	spi_4	Maha Sarakham	16.245500, 103.24900
	spi_5	Nakhon Ratchasima	14.560167, 101.97511
	spi_6	Uthai Thani	15.598611, 99.36611
	spi_7	Bangkok	13.844444, 100.57083
	spi_8	Samut Songkhram	13.474167, 99.97278
Wolffia globosa	wolf_3	Maha Sarakham	16.245500, 103.24900
	wolf_4	Bangkok	13.851139, 100.74047
	wolf_5	Surin	15.183861, 103.91186
	wolf_6	Bangkok	13.844444, 100.57083
	wolf_7	Lop Buri	14.924861, 100.90022
	wolf_8	Suphan Buri	14.166917, 99.94892
	wolf_9	Bangkok	13.688667, 100.65919

Table S6 Published data on duckweed growth traits. Relative growth rate (RGR, day^{-1}) and doubling time (days) of selected strains of *Landoltia punctata*, *Lemna aequinoctialis*, *Spirodela polyrhiza*, and *Wolffia globosa* from different geographic origins, as reported by Ziegler et al. (2015).

Species	Origin	RGR (frond/frond/day)	Doubling Time (days)
Landoltia punctata	China, Sichuan	0.388 ± 0.025	1.79 ± 0.15
	Ecuador, Esmeralde	0.380 ± 0.026	1.82 ± 0.13
	India, Delhi	0.365 ± 0.008	1.90± 0.04
	Australia, NSW	0.399 ± 0.012	1.74 ± 0.05
Lemna aequinoctialis	USA, California	0.476 ± 0.015	1.46 ± 0.05
	Venezuela	0.478 ± 0.025	1.47 ± 0.08
	India, AP	0.473 ± 0.016	1.47 ± 0.05
Spirodela polyrhiza	Puerto Rico	0.168 ± 0.011	4.13 ± 0.29
	USA, North Carolina	0.306 ± 0.005	2.27 ± 0.04
	Ecuador, Guayas	0.386 ± 0.025	1.80 ± 0.40
	Germany, Jena	0.328 ± 0.007	2.11 ± 0.05
	Albania, District Fieri	0.302 ± 0.012	2.30 ± 0.09
	India, Rajasthan	0.264 ± 0.012	2.63 ± 0.12
	India, Delhi	0.304 ± 0.004	2.28 ± 0.01
Wolffia globosa	China, Wuhan	0.328 ± 0.022	2.11 ± 0.14
	India, Delhi	0.457 ± 0.003	1.52 ± 0.01
	China, Sichuan	0.386 ± 0.047	1.80 ± 0.17
	China, Sichuan	0.369 ± 0.047	1.88 ± 0.24
